# Genetic Diversity and Population Structure of *Rumex crispus* in South Korea Based on Genome-Derived Microsatellite Markers

**DOI:** 10.3390/plants14243806

**Published:** 2025-12-14

**Authors:** Eun-Hye Kim, Kang-Rae Kim, Yujin Hwang, Ju-Hui Jeong, Jaeduk Goh, Jeong-Nam Yu, Mi-Hwa Lee

**Affiliations:** 1 Nakdonggang National Institute of Biological Resources, Sangju 37242, Republic of Korea; karpo83@nnibr.re.kr (E.-H.K.); dbwls8@nnibr.re.kr (Y.H.); juhii22@nnibr.re.kr (J.-H.J.); jdgoh@nnibr.re.kr (J.G.); susia000@nnibr.re.kr (J.-N.Y.); 2 Southeast Sea Fisheries Research , National Institute of Fisheries Science, Namhae 52440, Republic of Korea; kimkangrae9586@gmail.com

**Keywords:** *Rumex crispus*, microsatellite, genetic diversity, gene flow, population structure

## Abstract

*Rumex crispus* L. is a globally distributed invasive species that has naturalized in South Korea, where its use as a medicinal, edible, and ecological restoration resource continues to expand. However, its genetic background remains insufficiently understood, underscoring the need for species-specific molecular markers to enable accurate assessments of intraspecific genetic diversity and population structure. Using 19 newly developed microsatellite markers, we analyzed 120 plants from 6 populations in the riparian zone. A total of 166 alleles were detected, with a mean polymorphism information content of 0.637. Across the six populations, genetic diversity analysis showed mean observed (Ho = 0.304) and expected (He = 0.588) heterozygosity values indicative of heterozygote deficiency (inbreeding coefficient F_IS_ = 0.456–0.559). Genetic differentiation was low in AMOVA (10%) and F_ST_ (0.048–0.120) but higher in Jost’s D (0.096–0.342). STRUCTURE analysis identified two major genetic clusters (ΔK = 2), and spatial Bayesian clustering revealed six distinct genetic units (K = 6), suggesting that partial barriers to gene flow may have influenced population structure. These findings provide essential genetic insights that can support the effective control of *R. crispus* spread and its potential use as a valuable plant resource.

## 1. Introduction

Invasive species adapt flexibly to environmental conditions for reproduction and survival, enabling them to establish in new regions. They possess ecological strategies that facilitate rapid spread and persistence across diverse habitats [1]. Although the distribution of invasive species is not always constrained by climatic conditions, most regions where they become established exhibit environmental conditions similar to their native ranges [2]. Their invasion success is influenced by complex interactions with anthropogenic disturbances [3].

Once establishment is successful, invasive species form and maintain stable populations, reach high densities and abundances, and occupy key ecological positions within ecosystems [4,5]. This suggests that invasive species perform important ecological roles through competition or interaction with native species within existing ecosystems and may even engage in co-evolutionary relationships [6,7,8].

In some cases, invasive species exhibit higher genetic diversity in introduced ranges than in their native ranges, which has been attributed to admixture among individuals from repeated introductions [9,10]. Thus, the genetic background accumulated during establishment is closely linked to the species’ capacity to overcome bottlenecks and tolerate environmental stress [11].

*Rumex crispus* is native to Europe and is a naturalized invasive plant distributed globally, including in North America and Asia [12,13]. This species is perennial and is commonly found along roadsides, riverbanks, farm-lands, and gap or disturbed areas [14,15]. It flowers from June to July and is an anemophilous species pollinated by wind. It is also known to be capable of self-fertilization. A single plant produces several hundred to several thousand non-dormant seeds, and its growth varies substantially with soil fertility [16,17], reflecting strong physiological responsiveness to environmental conditions. Owing to its broad ecological niche and high survival and dispersal capacity, *R. crispus* has been designated as a noxious or managed species in several countries [18].

In South Korea, it is classified as a naturalized or alien species [19,20]. Although international plant databases [21] and some domestic scholars have suggested the possibility of post-glacial nativity [22], national specialized institutions estimate that the species was introduced either between the open-port period and 1921 [20] or after 1945 [15].

*R. crispus* has been utilized and studied not only as an edible plant but also as a resource possessing various pharmacological effects, including antioxidant, immune-response–suppressing, and antimicrobial activities [23,24,25]. Due to its rapid growth and the accumulation of pollutants in its aboveground tissues, the species has also been evaluated as a potential material for ecological restoration [26]. These aspects indicate that *R. crispus* serves a dual role, both as a species requiring management due to its excessive spread and as a valuable bioresource. Specifically, secondary metabolites are known to vary substantially within species, and this variation is strongly shaped by underlying genetic differences among individuals and populations [27,28]. Therefore, evaluating the genetic diversity of *R. crispus* is essential for understanding potential differences in its functional or bioactive traits.

Microsatellite (SSR) markers have been developed for *R. alpinus* [29] and *R. bucephalophorus* [30]. However, the genus *Rumex* is characterized by extensive cytogenetic variation, with multiple ploidy levels reported both among species and within species across different regions, often accompanied by interspecific hybridization [31]. Likewise, *R. crispus* shows regional variation in ploidy [32].

Although SSR markers exhibit limited reproducibility in polyploid taxa—primarily due to allele-dosage uncertainty—they remain highly informative due to their multi-allelic nature and strong discriminatory power [33]. Nonetheless, the species-specific nature of SSR loci highlights the need for marker development tailored specifically to *R. crispus*.

Accordingly, we aimed to develop microsatellite markers based on the whole-genome data of *R. crispus* in South Korea. We subsequently aimed to analyze genetic diversity by applying the developed markers to populations distributed across the country. The objective of this study is to provide fundamental data for future international comparative studies, as well as for regional genetic monitoring and bioresource utilization.

## 2. Results

### 2.1. Variation in Microsatellite Loci

A total of 19 microsatellite loci were successfully amplified and analyzed across all sampled individuals (Table 1). The polymorphism information content (PIC) values ranged from 0.387 (RcMS21) to 0.850 (RcMS26), with a mean of 0.637, indicating generally high levels of polymorphism. Across all loci, 166 alleles were detected, ranging from 2 to 9 per locus, with a mean of 4.9. The mean observed heterozygosity (Ho) was 0.265, whereas the mean expected heterozygosity (He) was 0.678, indicating that the observed heterozygosity was generally lower than expected. The mean inbreeding coefficient (F_IS_) was 0.591, indicating an overall heterozygote deficiency. Locus-specific F_IS_ values ranged from 0.078 (RcMS13) to 0.830 (RcMS70). Such positive F_IS_ values reflect heterozygote deficiency, which may be attributed to high levels of inbreeding within populations, whereas negative values indicate localized heterozygote excess. The overall mean F_ST_ was 0.106, indicating moderate genetic differentiation among populations. Locus-specific F_ST_ values varied substantially, ranging from 0.058 (RcMS03) to 0.184 (RcMS23), suggesting that certain markers contributed disproportionately to population divergence.

### 2.2. Genetic Diversity Analysis

Genetic diversity was analyzed across 6 populations using 19 microsatellite loci. A total of 166 alleles were detected across all populations. The observed number of alleles per population ranged from 71 (YD) to 103 (GJ and DJ), with a mean of 89.8 (Table 2).

**Figure 1:**
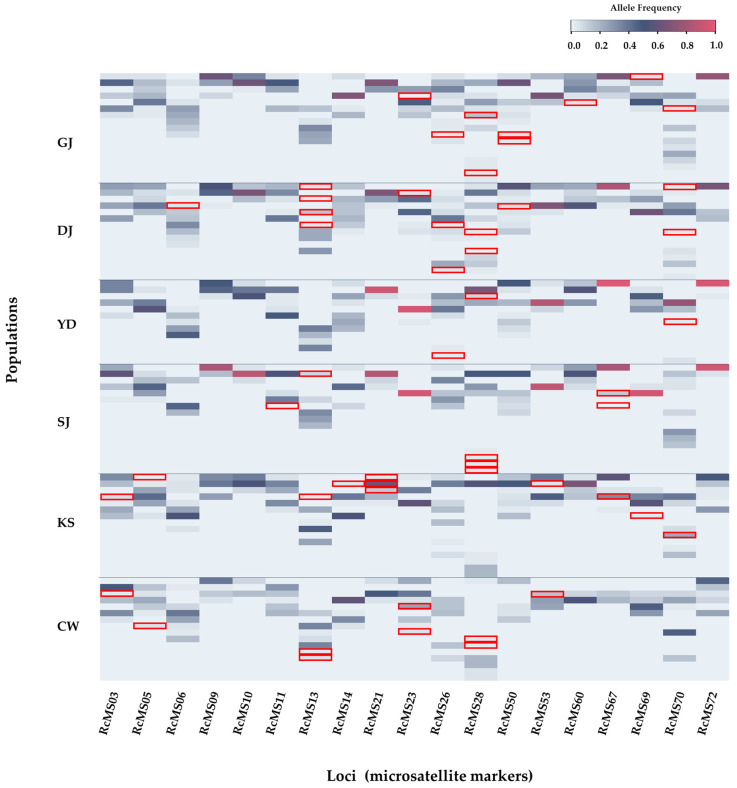
Heatmap showing common and unique allele frequency distributions across six *Rumex crispus* populations (GJ, DJ, YD, SJ, KS, and CW) based on 19 microsatellite loci. Each cell color represents the relative frequency of a particular allele (blue-gray = 0, red = 1). Red boxes: locus of unique alleles.

Unique alleles were identified at 16 loci, each containing 1–10 such alleles, totaling 52 unique alleles (Figure 1). The DJ population exhibited the highest number of unique alleles, with 13 alleles found across 7 loci, whereas the YD population had only 3 unique alleles, each from a different locus; in addition, 1 locus showed fixation of alleles.

The proportion of polymorphic loci (P_0.95_) was 100% in all populations, except in YD, which showed a slightly reduced value of 89.5% due to the presence of a fixed allele (Table 2). The effective number of alleles per locus (Ae/L) varied from 2.4 (YD and SJ) to 3.4 (CW), with an average of 2.9. Observed heterozygosity (Ho) ranged from 0.258 (SJ) to 0.355 (DJ), with a mean of 0.304, whereas expected heterozygosity (He) ranged from 0.472 (SJ) to 0.680 (CW), with a mean of 0.588. All individuals represented distinct multilocus genotypes (MLGs).

At the species level, the He value was higher than the Ho value, indicating the possibility of a genetic bottleneck in some populations. The inbreeding coefficient (F_IS_) for each population indicated heterozygosity deficiency, ranging from 0.456 (DJ) to 0.559 (KS) (Table 3). The bottleneck test under the Infinite Allele Model (IAM) showed significant results for all populations except SJ. Under the Two-Phase Mutation Model (TPM), a strong bottleneck signal was detected in the CW population. No statistically significant results were obtained under the Stepwise Mutation Model (SMM) for any population.

### 2.3. Genetic Differentiation and Gene Flow

**Figure 2:**
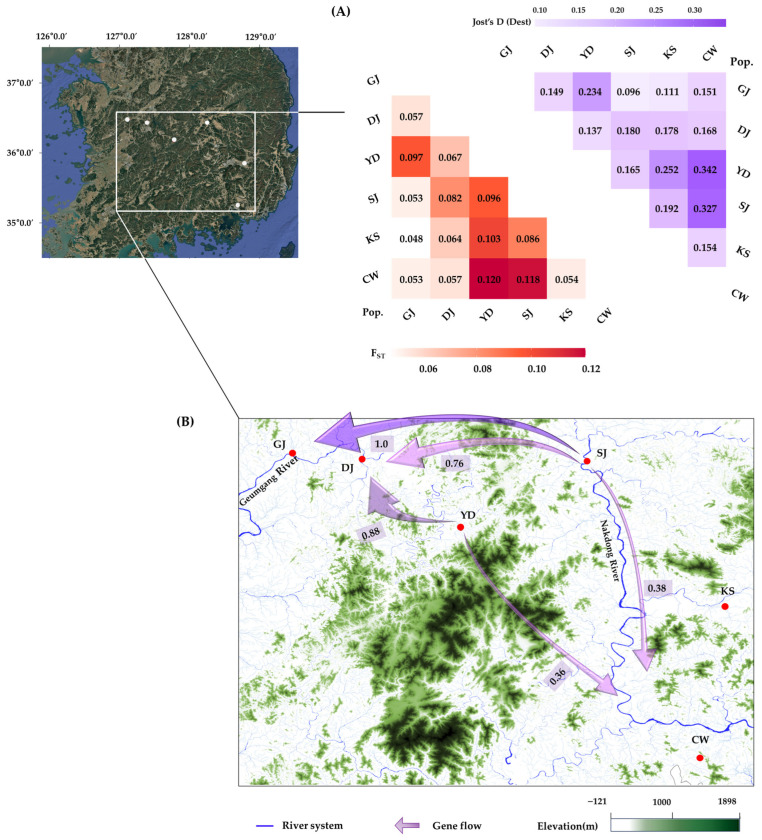
Genetic differentiation and directional gene flow among *R. crispus* populations in South Korea. (**A**) Pairwise F_ST_ values (below diagonal, red gradient) and Jost’s D (Dest) values (above diagonal; purple gradient) among six populations; (**B**) Geographic distribution of populations and inferred directional gene flow patterns (Scale 1: 550,000).

Genetic differentiation among the six populations was 10%, with 90% of the genetic variance occurring within populations. Pairwise F_ST_ values ranged from 0.048 (between GJ and KS) to 0.120 (between YD and CW). Jost’s D (Dest) revealed a broader spectrum of differentiation, ranging from 0.096 (between SJ and GJ) to 0.342 (between YD and CW), indicating stronger differentiation than that reflected by F_ST_ values (Figure 2A).

Directional migration analysis (Figure 2B) was conducted to assess the directionality of gene flow among the six sampling sites. Most relative migration estimates were moderate, suggesting a limited degree of allele exchange, and overall connectivity among populations remained low. Directional patterns revealed a relatively consistent unidirectional flow from SJ toward GJ, DJ, and CW, with YD also contributing gene flow of alleles to DJ and CW. In contrast, KS exhibited restricted gene flow and appeared more isolated from the other populations. Furthermore, a Mantel test revealed a weak but statistically significant positive correlation between genetic and geographic distances (*r* = 0.189, *p* < 0.001), indicating an isolation-by-distance (IBD) pattern among the populations.

### 2.4. Spatial and Genetic Structure

**Figure 3:**
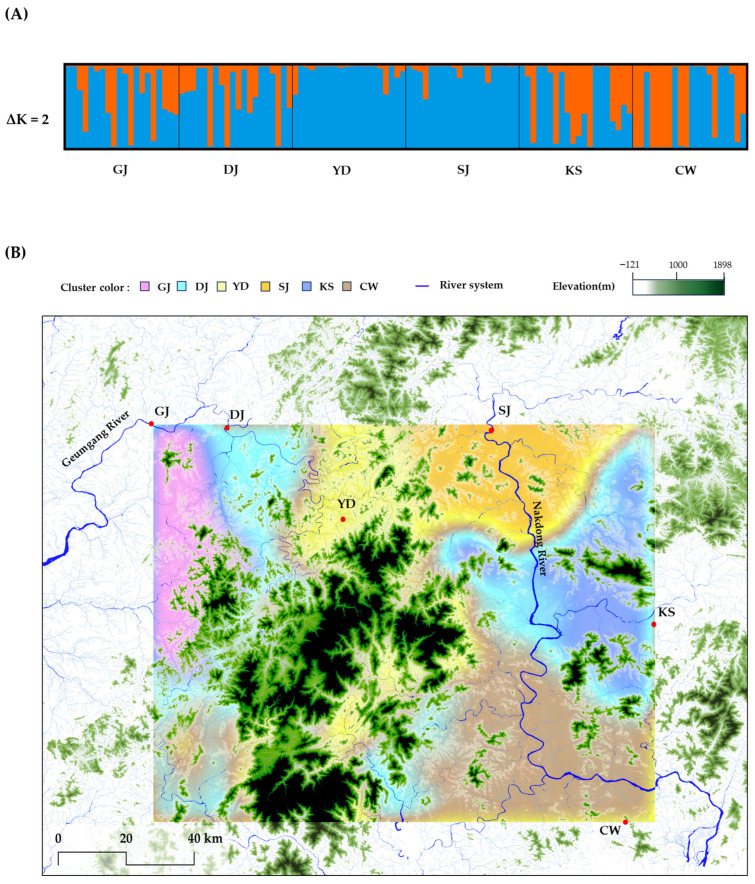
Spatial genetic structure of *R. crispus* populations. (**A**) STRUCTURE bar plots showing allele frequency–based clustering results (ΔK = 2), and (**B**) spatially explicit genetic clusters inferred from GENELAND (K = 6), displayed as color-coded background layers. (Scale = 1:650,000).

Genetic structure analysis of the six populations indicated an optimal number of clusters (ΔK = 2) (Figure S2), dividing the populations into two major genetic groups (Figure 3A). The YD and SJ populations were almost exclusively associated with cluster 1 (>94%). The GJ and DJ populations were also mainly assigned to cluster 1 (0.63–0.68), although they showed partial membership in cluster 2. The KS population was similarly associated with cluster 1, whereas the CW population was assigned to cluster 2.

The spatial (geographic) Bayesian clustering analysis (K = 6) revealed that each population formed an independent genetic cluster, and distinct genetic barriers were detected among populations (Figure 3B).

## 3. Discussion

### 3.1. Variation in Microsatellite Loci

The 19 microsatellite loci developed in this study exhibited a wide range of PIC values, from 0.387 to 0.850 (Table 1). The mean PIC value (0.637) indicated that most loci were sufficiently informative for population genetic analysis. A total of 166 alleles were detected across all loci, with each population possessing 3–13 unique or rare alleles.

The mean F_IS_ value across all loci was relatively high (0.591), and most loci significantly deviated from Hardy–Weinberg equilibrium (HWE). All analyzed individuals possessed MLGs. The observed heterozygote deficiency was therefore likely caused by inbreeding effects, consistent with the mixed reproductive system previously reported for *R. crispus* [16]. These results suggest that the observed deviations among loci reflect the species’ reproductive strategy and mating system. Therefore, the microsatellite markers developed in this study can serve as reliable molecular tools for elucidating genetic diversity, population differentiation, and reproductive characteristics in *R. crispus*.

### 3.2. Genetic Diversity Analysis

The mean values of observed (Ho = 0.304) and expected heterozygosity (He = 0.588) of *R. crispus* in this study were higher than those reported for the congeneric species *R. alpinus* (Nei’s gene diversity, He = 0.43; [34]). Although we attempted to compare genetic diversity values among studies of the same species or genus, direct comparisons were limited due to differences in marker types and analytical methods. When compared with other perennial herbaceous species exhibiting invasive characteristics and analyzed using microsatellite markers, *R. crispus* showed slightly lower or comparable levels of genetic diversity to those of *Ambrosia artemisiifolia* (Ho = 0.544, He = 0.630 [35]); *A. psilostachya* (He = 0.43 ± 0.13 [36]); *Senecio madagascariensis* (Ho = 0.456, He = 0.715 [37]); and *Silene latifolia* (He = 0.462–0.668 [38]).

Among the six populations, SJ (Ho = 0.258, He = 0.472) showed lower genetic diversity than the other five populations (Table 2). These patterns may be related to population reproductive dynamics or local habitat conditions, suggesting the need for ecological monitoring to better understand the underlying factors.

When an invasive species is first introduced, its populations often experience genetic drift, founder effects, and increased inbreeding resulting from population bottlenecks [10,39]. The CW population, located at the geographic margin, showed higher genetic diversity than the population average; nevertheless, a bottleneck signal was detected. Notably, F_IS_ values around or above 0.5 indicated substantial inbreeding pressure in these populations (Table 3).

This apparently paradoxical genetic pattern can be explained by three possible scenarios [40,41]. First, it may represent post-bottleneck recovery, wherein genetic diversity was rapidly restored through gene flow and sexual reproduction following a temporary reduction in effective population size [42,43]. Second, it may reflect recolonization, where continuous immigration from external populations led to genetic admixture after disturbance events [44]. Third, a loss of rare alleles (frequency < 0.025) while retaining common alleles could produce detectable bottleneck signals even under high heterozygosity levels [45,46].

Given the sensitivity of the TPM to short-term demographic fluctuations, the bottleneck signal observed in the CW population may reflect recent demographic instability rather than an actual loss of genetic variation (Figure S1) [47]. These findings likely correspond to fluctuations associated with recent habitat changes. Keller and Waller (2002) [48] suggested that this process could result in increased among-population heterosis, as crosses between genetically differentiated populations may restore fitness lost through inbreeding [49,50]. Although our data do not directly test this hypothesis, the findings indicate a possible evolutionary scenario wherein drift-driven divergence contributes to the long-term persistence of *R. crispus* populations despite demographic instability.

### 3.3. Genetic Differentiation and Gene Flow

The genetic differentiation of *R. crispus* (Φ_ST_ = 0.10) indicates relatively active gene flow among regions compared with related or ecologically similar species such as *R. alpinus* (Φ_ST_ = 26.8%; [34]), *A. artemisiifolia* (F_ST_ = 0.064; [35]), *A. psilostachya* (Φ_ST_ = 10.4%; [36]), *S. madagascariensis* (F_ST_ = 0.103; [37]), and *S. latifolia* (F_ST_ = 0.179; [38]). However, pairwise comparisons revealed pronounced genetic differentiation among populations (Figure 2A). In addition, the correlation between geographic distance and genetic distance was weak (*r* = 0.189, *p* < 0.001), indicating that gene flow exists but does not follow a consistent or predictable spatial pattern. The relatively low overall genetic differentiation, despite the high inbreeding coefficients observed in each population (F_IS_ = 0.456–0.559), should therefore be interpreted in the context of the species’ biological and ecological characteristics [42].

Gene flow has long been recognized as a key factor shaping population structure and maintaining connectivity among populations [51]. More recently, it has also been examined for its role in range expansion and adaptive differentiation [52]. The directional pattern of gene flow observed in this study exhibited a distinct trend (Figure 2B). The influence of hydrological factors on genetic connectivity appeared relatively weak among populations within the same watershed (i.e., GJ, DJ, and YD in the Geum River watershed, and KS and SJ in the Nakdong River watershed). Notably, the SJ population acted as a donor, contributing gene flow to the GJ and DJ populations located in a different watershed, independently of the KS population within the same watershed. These results suggest that factors other than hydrological connectivity may have contributed to the observed gene flow patterns.

The Nakdong River, where the SJ population is located, is one of the major rivers in South Korea, and its sediments have long been utilized as high-quality construction aggregates [53,54]. Considering the long-term viability of buried seeds, often persisting for several decades [12,55], it is plausible that propagules or genetic materials such as seeds and root fragments from the SJ population were incorporated into dredged sediments during these activities. Consequently, such propagules may have been dispersed and established in other regions, thereby contributing to the regional gene pool. Thus, gene flow and genetic differentiation together likely shaped the overall genetic structure among populations.

### 3.4. Spatial and Genetic Structure

The population genetic structure inferred from allele frequency data revealed two genetic clusters, reflecting gene flow among populations. However, when geographic positions were considered, each population exhibited a distinct spatial genetic structure (Figure 3). This pattern was consistent with the results of pairwise genetic differentiation (Figure 2A) and the Mantel test (r = 0.189, *p* < 0.001). Although genetic differentiation associated with geographic distance was relatively low, the existence of genetic barriers among populations is likely influenced by mating among genetically related individuals within regions, as well as by the presence of unique alleles in certain populations. These findings suggest that anthropogenic factors contribute to the formation of spatial genetic structure. When interpreted alongside genetic diversity data, the observed genetic structure likely reflects the influence of habitat conditions.

*R. crispus* is sensitive to soil moisture and nutrient availability, which can directly affect its growth and reproductive success [13,18]. Eutrophic water conditions along riparian zones in South Korea (Table S2) may have promoted rapid growth and facilitated recolonization through non-dormant seeds [56,57] capable of germinating quickly under favorable conditions [12,58], which likely contributed to the widespread establishment across diverse habitats.

Although *R. crispus* is a common and widely distributed species rather than a rare or endangered one, it has garnered relatively little research interest. Due to the limited number of sampling sites, this study may not fully represent the genetic patterns of *R. crispus* populations across the entire South Korean Peninsula. Nevertheless, the newly developed microsatellite markers provide high resolution for detecting fine-scale genetic structures. These findings are expected to inform management strategies and resource utilization for invasive plants, not only in South Korea but also in other regions with similar habitat conditions.

## 4. Materials and Methods

**Figure 4:**
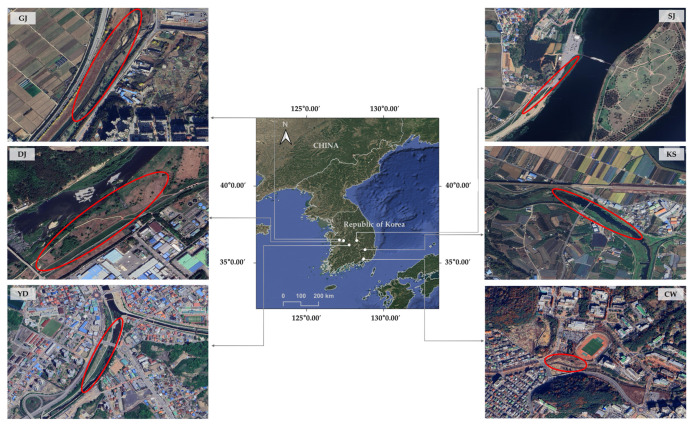
Geographic distribution of six *R. crispus* populations (red circle) across South Korea and corresponding satellite images of each sampling site (GJ, DJ, YD, SJ, KS, and CW). (Scale 1: 3000).

In June 2023, 120 individual plant units (20 per population) of *Rumex crispus* were collected from 6 populations inhabiting riparian zones across South Korea (Figure 4).

To minimize the likelihood of sampling genetically related plants resulting from limited mating or pollen dispersal, each sample was collected at least 10 m apart. The sampling sites were classified into three hydrological systems. Three populations—GJ (36°28′46.1″ N, 127°07′55.8″ E), DJ (36°21′51.3″ N, 127°23′42.9″ E), and YD (36°10′12.3″ N, 127°46′26.4″ E)—belonged to the Geum River Basin. Two populations—SJ (36°26′31.3″ N, 128°15′20.1″ E) and KS (35°51′01.8″ N, 128°47′12.7″ E)—were part of the Nakdong River Basin. The remaining population—CW (35°14′32.7″ N, 128°41′28.6″ E)—was located at the confluence of river and coastal currents, representing an independent hydrological system connected to the lower Nakdong River and adjacent coastal areas. All voucher specimens have been deposited in the Nakdonggang National Institute of Biological Resources (NNIBR), South Korea (Voucher Nos. NNIBRVP126595–126734).

### 4.1. Microsatellite Marker Development and Functional Genomic Analysis

Silica gel-dried leaf tissues were stored at −80 °C until use. Genomic DNA was extracted using the DNeasy Plant Mini Kit (QIAGEN, Hilden, Germany) according to the manufacturer’s instructions. Microsatellite regions were identified from the whole genome assembly (NCBI BioSample: SAMN50897494, JBQWDL000000000) using the Microsatellite (MISA) tool (https://webblast.ipk-gatersleben.de/misa/, accessed on 12 December 2023). Screening parameters were set to detect a minimum of 10 repeats for dinucleotide motifs and at least 4 repeats for tri-, tetra-, penta-, and hexanucleotide motifs. Primers were designed using Primer3 (https://github.com/primer3-org/primer3, accessed on 12 December 2023) with the following parameters: amplicon size of 100–300 bp, primer length of 20–24 bp, GC content of 40–60%, and melting temperature of 58 °C. Primer specificity was confirmed using SnapGene (GSL Biotech, Chicago, IL, USA) to ensure that each primer pair uniquely bound to the target sequence. A total of 100 primer pairs were initially designed. PCR conditions were optimized, and each primer was tested on six plants per population to assess polymorphism. Nineteen polymorphic and reproducibly amplified loci were selected for further analysis of all samples (Table 1). The final set of 19 validated microsatellite loci was submitted to GenBank, and accession numbers PX048371-PX048381, PX048383-PX048389, and PX055712 (RcMS11) are provided.

Microsatellite locus amplification was performed using the Mastercycler^®^ Pro Gene Amplifier (Eppendorf, Hamburg, Germany). PCR was conducted at a total volume of 20 μL using H-Star Taq DNA Polymerase (Biofact, Daejeon, Republic of Korea). Fluorescent labeling followed the protocol described by Schuelke [59], employing four fluorescent dyes (6-FAM, VIC, NED, PET) (Supplementary Figure S3). Each reaction mixture contained a locus-specific forward primer (0.4 μM), a reverse primer with an M13 tail sequence (5′-TGTAAAACGACGGCCAGT, 0.8 μM), and a fluorescently labeled M13 primer (0.4 μM). PCR cycling conditions consisted of an initial denaturation at 94 °C for 5 min; 30 cycles of denaturation at 94 °C for 30 s, annealing at 60 °C for 45 s, and extension at 72 °C for 45 s; followed by 12 cycles at 94 °C for 30 s, 53 °C for 45 s, and 72 °C for 45 s. A final extension was performed at 72 °C for 10 min. PCR products were mixed with GeneScan™ 500 ROX Size Standard Ladder (Applied Biosystems, Foster City, CA, USA) and HiDi™ formamide, denatured at 95 °C for 2 min, and cooled to 4 °C. Allele sizing was conducted using an ABI 3730xl DNA Analyzer (Applied Biosystems), and genotyping was performed using GeneMarker^®^ ver. 2.6.7 (SoftGenetics, State College, PA, USA).

### 4.2. Genetic Diversity Analysis

MICROCHECKER ver. 2.2.3 [60] was used to detect potential scoring errors, including stuttering, large-allele dropout, and null alleles, based on deviations from expected heterozygote frequencies and repeat-motif patterns (1000 Monte Carlo iterations, 95% CI). PIC values were estimated using Cervus ver. 3.0.7 [61], whereas F-statistics were derived following Nei’s method implemented in FSTAT ver. 2.9.4 [62]. To visualize the distribution of common and population-specific alleles across the 19 microsatellite loci, a heatmap was generated using the Heatmapper web tool (http://www.heatmapper.ca/expression/:accessed on 21 September 2025) based on allele frequency data from each sampling site.

Genetic diversity parameters, including the percentage of polymorphic loci (P_0.95_), the mean number of alleles per locus (A), the effective number of alleles per locus (Ae), and observed (Ho) and expected (He) heterozygosity, were calculated using Popgene v1.32 [63]. Recent bottleneck events were tested using BOTTLENECK v1.2.02 [64]. Three mutation models—the IAM, SMM, and TPM—were applied. The TPM (70% SMM, 30% IAM, variance = 12) served as the primary model. Significance was evaluated using the Wilcoxon signed-rank test (one-tailed) based on 10,000 iterations. Mode-shift indicator graphs were also generated to visualize allele frequency distributions across populations (Supplementary Figure S1).

### 4.3. Genetic Differentiation and Gene Flow

To determine genetic differentiation among populations, Wright’s F-statistics (F_IS_, F_IT_, F_ST_; [65]) were calculated using FSTAT ver. 2.9.4 [62]. F_IS_ values were calculated for polymorphic loci only, whereas loci with a dominant allele frequency ≥ 0.95 were treated as fixed and excluded from the estimation. Their significance was assessed by permutation tests with 180,000 randomizations. To assess population differentiation, the statistical significance of F_ST_ and Jost’s D (D_est)_ [66] was evaluated by calculating the 95% confidence intervals, with 1000 permutations and bootstraps using GenAIEx ver. 6.503 [67].

The correlation between genetic differentiation and geographic distance among populations was examined using Mantel tests [68] conducted in the IBD software ver. 1.52 [69]. Directional gene flow (D) and asymmetric migration were analyzed using the DivMigrate online application [70], developed as part of the diveRsity R package [71], with D as the differentiation metric and 1000 bootstrap replicates.

### 4.4. Spatial and Genetic Structure

To investigate the genetic structure of each population, STRUCTURE ver. 2.3.4 [72] was employed to identify discrete genetic clusters and estimate the proportion of individual genotypes assigned to each cluster. Simulations were run 10 times for each ΔK value (1–10), with 1,000,000 MCMC iterations following a burn-in of 100,000, using the admixture model and assuming correlated allele frequencies [73,74]. The optimal number of clusters (K) was inferred using the Structure Selector web tool [75]. To correct for label switching among replicate STRUCTURE runs, Q-matrices were aligned and averaged using CLUMPP (Greedy algorithm) to obtain a consensus cluster assignment used for final visualization.

To characterize spatial patterns of genetic variation in *R. crispus*, cluster analysis was conducted in GENELAND v4.9.2 [76]. The number of clusters (K) was tested from 1 to 6, reflecting the sampled populations. Each analysis consisted of 1,000,000 MCMC iterations with a thinning interval of 500, and was replicated 10 times to ensure convergence and consistency across independent runs. The resulting spatial clusters (K = 6) of population membership maps were visualized in QGIS v3.34 (QGIS Development Team, 2023).

## 5. Conclusions

In this study, we analyzed the genetic diversity and population genetic structure of *R. crispus* populations in South Korea using newly developed microsatellite markers. Despite the limited number of sampled populations, allelic variation within each population revealed detectable genetic diversity and structural distinctiveness. The *R. crispus* populations in South Korea exhibited considerable levels of genetic diversity; however, all populations showed heterozygote deficiency resulting from inbreeding. Considering the intensified genetic differentiation among populations and its inconsistency with the observed geographic distribution patterns, the results of directional gene flow analysis indicate that connectivity among populations is asymmetric and follows patterns that cannot be explained solely by geographic distance or watershed structure.

The coexistence of bottleneck signals and relatively high genetic diversity in certain populations likely reflects short-term demographic fluctuations and recent habitat disturbances, indicating a strong capacity for recovery.

Given the ecological tolerance of *R. crispus* to diverse environmental conditions, management practices such as mowing before the reproductive or flowering stages could effectively suppress uncontrolled spread. Furthermore, the potential for inbreeding may help maintain unique genetic characteristics within local populations, which could serve as valuable sources of region-specific bioactive or pharmacological compounds. Therefore, management strategies should not only focus on controlling its spread but also explore the potential utilization of its genetic and physiological resources.

In the future, extended studies incorporating habitat types, ecological factors, and transcriptome-based genetic analyses will be essential to gain a deeper understanding of the adaptive potential of *R. crispus* under changing climate conditions.

## Figures and Tables

**Table 1:** Assessment of genetic variations of 19 microsatellite loci developed from *Rumex crispus*.

Genbank Accession No.	Locus	PIC	A/L	Ae/L	Ho	He	F_IS_	F_ST_	HWE
PX048371	RcMS03	0.719	4	1.96	0.625	0.763	0.138	0.058	***
PX048372	RcMS05	0.715	7	1.88	0.658	0.756	0.067	0.079	***
PX048373	RcMS06	0.794	4	2.75	0.258	0.823	0.660	0.092	***
PX048374	RcMS09	0.503	7	3.73	0.167	0.591	0.690	0.106	***
PX048375	RcMS10	0.490	9	2.77	0.200	0.553	0.605	0.100	***
PX055712	RcMS11	0.706	5	3.66	0.258	0.750	0.626	0.093	***
PX048376	RcMS13	0.772	3	1.96	0.692	0.804	0.078	0.079	***
PX048377	RcMS14	0.700	3	2.63	0.192	0.743	0.712	0.123	***
PX048378	RcMS21	0.387	5	2.90	0.142	0.446	0.654	0.096	***
PX048379	RcMS23	0.505	2	1.13	0.150	0.560	0.682	0.184	***
PX048380	RcMS26	0.850	4	2.57	0.400	0.870	0.503	0.089	***
PX048381	RcMS28	0.738	4	3.02	0.317	0.756	0.550	0.082	***
PX048383	RcMS50	0.849	6	2.25	0.142	0.870	0.825	0.083	ND
PX048384	RcMS53	0.477	3	1.34	0.092	0.532	0.809	0.116	***
PX048385	RcMS60	0.668	8	5.57	0.242	0.721	0.624	0.127	***
PX048386	RcMS67	0.406	3	2.02	0.108	0.432	0.726	0.101	***
PX048387	RcMS69	0.584	6	1.67	0.158	0.624	0.710	0.146	***
PX048388	RcMS70	0.803	6	4.46	0.125	0.828	0.830	0.132	***
PX048389	RcMS72	0.429	5	3.46	0.108	0.461	0.738	0.122	***
	Mean	0.637	4.9	2.72	0.265	0.678	0.591	0.106	

PIC: Polymorphism information content; A/L: Number of alleles per locus; Ae/L: Number of effective alleles per locus; Ho: Observed heterozygosity; He: Expected heterozygosity; F_IS_: Inbreeding coefficient; F_ST_: Genetic differentiation at each locus; HWE: Hardy–Weinberg equilibrium (*** *p* < 0.001).

**Table 2:** Genetic diversity in six populations of *R. crispus* analyzed by 19 microsatellite loci.

Population	N	P(_0.95_)	A	Ae/L	A_U_	Ho	He	G
GJ	20	100	103	3.2	9	0.332	0.633	20
DJ	20	100	103	3.2	13	0.355	0.629	20
YD	20	89.5	71	2.4	3	0.261	0.481	20
SJ	20	100	72	2.4	7	0.258	0.472	20
KS	20	100	92	2.9	11	0.290	0.631	20
CW	20	100	98	3.4	9	0.332	0.680	20
Mean	20	98.2	89.8	2.9	8.7	0.304	0.588	20
Total	20	100	166	3.6	52	0.304	0.669	120

N: Number of samples; P(_0.95_): Polymorphic loci were determined based on the 0.95 criterion; A: Observed number of alleles; Ae/L: Effective number of alleles per locus; Ho: Observed heterozygosity; He: Expected heterozygosity; G: Number of multi-locus genotypes; A_U_: Number of unique alleles.

**Table 3:** Results of the Wilcoxon signed-rank test for bottleneck detection under three mutation models (IAM, SMM, TPM), using one-tailed analyses (Hep). *p*-values of expected heterozygosity obtained from the Infinite Allele Model (IAM), Stepwise Mutation Model (SMM), and Two-Phase Mutation Model (TPM).

Population	He	IAM	SMM	TPM	F_IS_
GJ	0.633	0.566 **	0.686	0.616	0.496
DJ	0.629	0.572 **	0.670	0.623	0.456
YD	0.481	0.465 *	0.564	0.517	0.478
SJ	0.472	0.450	0.542	0.497	0.474
KS	0.631	0.560 **	0.666	0.617	0.559
CW	0.680	0.581 ***	0.686	0.637 **	0.531

Significance levels: (*p* < 0.05 *, *p* < 0.01 **, *p* < 0.001 ***); F_IS_ = Inbreeding coefficient.

## Data Availability

The original contributions of this study are included in the article. Further inquiries can be directed to the corresponding author.

## Supplementary Materials

The following supporting information can be downloaded at: https://www.mdpi.com/article/10.3390/plants14243806/s1, Table S1: Amplification information, primer sequences, and characteristics of 19 microsatellite loci developed from *Rumex crispus*. Table S2: Mean (±SD) values of major water quality parameters (DO, TN, and TP) at monitoring sites near each *R. crispus* population, based on data from 2011 to 2023 (source: Water Management Information System (WAMIS; https://www.wamis.go.kr: accessed on 13 October 2025), Ministry of Environment, Republic of Korea). Figure S1: Mode-shift distributions of allele frequency classes for the six *R. crispus* populations. Figure S2: Selection of the optimal number of genetic clusters (K) in STRUCTURE. ΔK plot based on the Evanno method, identifying K = 2 as the most likely number of clusters (left) and Mean LnP(K) ± Stdev across replicate runs, illustrating trends in model likelihood and variability for each K value; red dashed lines indicate results for K = 10 (right). Figure S3: Representative electropherograms of four SSR loci (RcMS06, RcMS28, RcMS70, and RcMS72). Fluorescently labeled PCR fragments were analyzed using an ABI capillary electrophoresis platform.
