# Supplementary material for: Genetic Diversity and Population Structure of Rumex crispus in South Korea Based on Genome-Derived Microsatellite Markers
Source: Plants (Basel). 2025 Dec 14;14(24):3806. doi: 10.3390/plants14243806 (PMC12736510; doi:10.3390/plants14243806)
Supplement: Supplementary file 1 [file plants-14-03806-s001.zip › plants-3994835-supplementary.pdf]

Supplementary

Table S1. Amplification information, primer sequences, and characteristics of 19 microsatellite loci developed from *Rumex crispus.*

Locus	Primer sequence (5′-3′)	Motif	Allele range (bp)	Label
RcMS03	F: AGTTGCAAGGGGAGGTGATG<br>R: TCCATGACCACCAAAGGCAT	(ATC)7	222-238	F
RcMS05	F: TTCCGGAGTACGTTGTTGGG<br>R: ACTTGCCTCGACAACTCGTT	(TTA)15	208-248	F
RcMS06	F: TCTATAATGCCACGGCCCTG<br>R: CTCGTCCCTAGTGATGCGAC	(CCT)15	207-233	F
RcMS09	F: ATCACAGGTGAAGGTTGCGT<br>R: CGGGAGACGGGAAGAAGATG	(TGT)10	218-236	F
RcMS10	F: CTGTCTAGGTTCGGCGTAGG<br>R: ACTGAAGAGGAGTGCACTCG	(TA)13	220-224	F
RcMS11	F: GGCTGGTTGACTGGGTGAA<br>R: GTGTAGACTTGGCCCCGATT	(TAT)13	205-322	F
RcMS13	F: TCGGAGGAATGCTTTCGGAG<br>R: ACTTGCCTCGACAACTCGTT	(TTA)15	217-254	F
RcMS14	F: TCACGTATACGCATGCCTCT<br>R: AATACGTGAATGCGTGAGGC	(AT)11	212-238	F
RcMS21	F: CGGCGCACTTTGTTCAAAGA<br>R: CGCCGAAGATGAAGAAAGCG	(CTCC)9	231-247	F
RcMS23	F: TGCACAATCACAAGCTCGTG<br>R: TAATCCACCCACCTGCATGG	(CT)13	224-250	F
RcMS26	F: ATCTACGCCGGATCTTCACG<br>R: AAAGTGACGTCGCAGGGAAT	(TC)12	216-276	F
RcMS28	F: GAGACCGCCACGAGTCTATG<br>R: CGGTACACTGTTCCTTGCCT	(CT)13	238-292	V
RcMS50	F: CGACAAATCAGCGATGTGGA<br>R: GAACAAAATGTGGTGGGCCC	(AT)22	236-272	V
RcMS53	F: CAACGTCGCCAGGAGTAGAA<br>R: TGCATGCTTGTAACCGGACT	(CT)13	254-264	V
RcMS60	F: AGAGTTTGGCACCATCTTACGT<br>R: AAGCTTTCCGATGCTTGGGA	(TA)10	270-278	N
RcMS67	F: TCCATCTCGGAAGCCTCCTA<br>R: TGTGTGGTGGATCATGGTTGT	(TA)12	270-312	N
RcMS69	F: CTCATGTTTTGGGCACGCAA<br>R: ACGCTCAGGTGAAACGGTAA	(CT)13	276-294	N
RcMS70	F: GCGAAAGGGGAATGCATGAC<br>R: AGAAGGCGTTTCAAGTGGATCT	(AT)13	280-322	N
RcMS72	F: ATCCGACACGATCCGAAGTG<br>R: TGGATTTGGCTGGCTTGCTA	(ATC)7	286-300	P

Label codes: F = FAM, V = VIC, N = NED, P = PET

Table S2. Mean (±SD) values of major water quality parameters (DO, TN, and TP) at monitoring sites near each *R. crispus* population, based on data from 2011-2023 (Source: Water Management Information System (WAMIS; https://www.wamis.go.kr), Ministry of Environment, Republic of Korea).

Population	DO	TN	TP
GJ	10.8 (±2.3)	3.0 (±1.1)	0.072 (±0.053)
DJ	10.0 (±1.8)	7.5 (±2.6)	0.156 (±0.136)
YD	12.3 (±2.3)	3.9 (±1.3)	0.020 (±0.030)
SJ	10.5 (±2.1)	2.3 (±0.6)	0.039 (±0.031)
KS	12.3 (±2.9)	3.5 (±1.4)	0.049 (±0.043)
CW	7.2 (±3.0)	2.7 (±1.4)	0.118 (±0.098)

DO: Dissolved Oxygen; TN: Total Nitrogen; TP: Total Phosphorus.

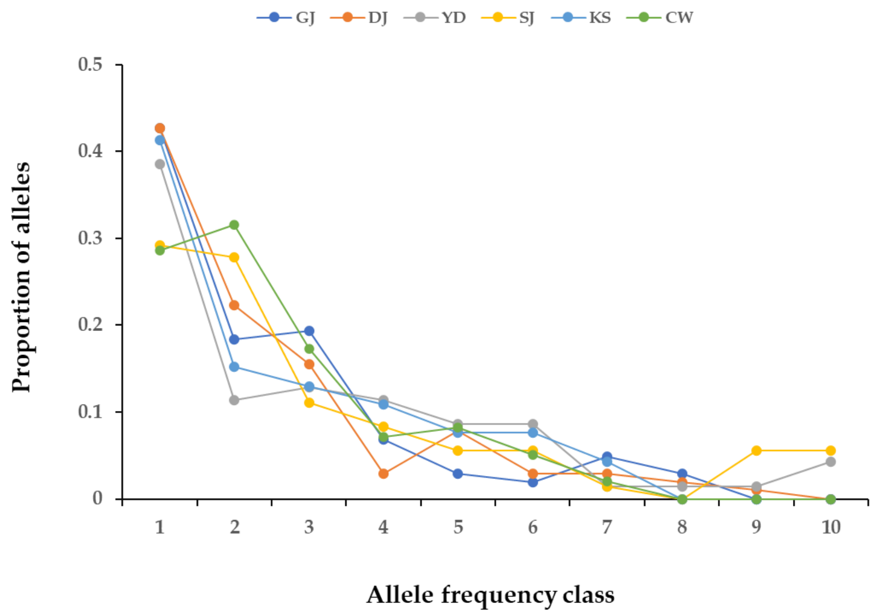

Figure S1. Mode-shift distributions of allele frequency classes for the six *R. crispus* populations.

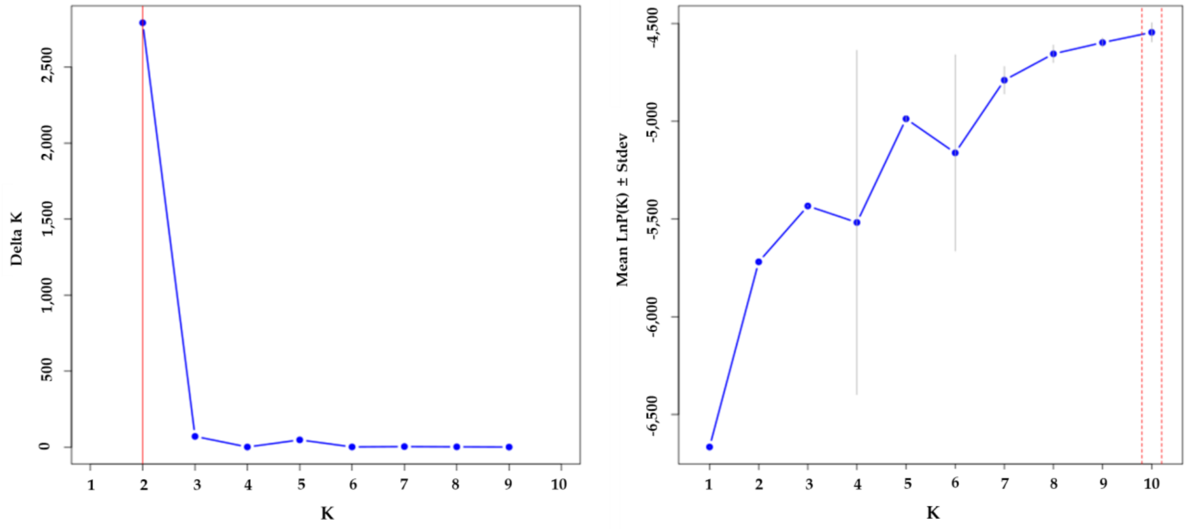

Figure S2. Selection of the optimal number of genetic clusters (K) in STRUCTURE. ΔK plot based on the Evanno method, identifying K = 2 as the most likely number of clusters (left), and Mean LnP(K) ± Stdev across replicate runs, illustrating trends in model likelihood and variability for each K value; red dashed lines indicate results for K = 10 (right).

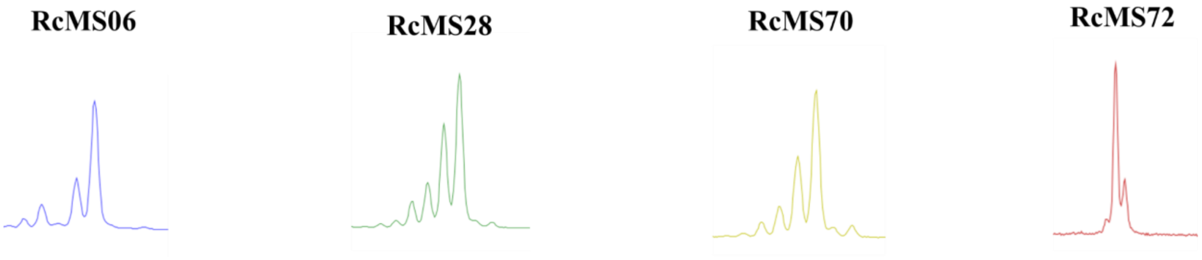

Figure S3. Representative electropherograms of four SSR loci (RcMS06, RcMS28, RcMS70, and RcMS72). Fluorescently labeled PCR fragments were analyzed using an ABI capillary electrophoresis platform.
